# Influenza Virus-Mediated Membrane Fusion: Determinants of Hemagglutinin Fusogenic Activity and Experimental Approaches for Assessing Virus Fusion

**DOI:** 10.3390/v4071144

**Published:** 2012-07-24

**Authors:** Brian S. Hamilton, Gary R. Whittaker, Susan Daniel

**Affiliations:** 1 Department of Microbiology and Immunology, Cornell University, Ithaca, NY 14853, USA; Email: bsh65@cornell.edu; 2 School of Chemical and Biomolecular Engineering, Cornell University, Ithaca, NY 14853, USA; Email: sd386@cornell.edu

**Keywords:** influenza virus, hemagglutinin, membrane fusion, protease, virus entry

## Abstract

Hemagglutinin (HA) is the viral protein that facilitates the entry of influenza viruses into host cells. This protein controls two critical aspects of entry: virus binding and membrane fusion. In order for HA to carry out these functions, it must first undergo a priming step, proteolytic cleavage, which renders it fusion competent. Membrane fusion commences from inside the endosome after a drop in lumenal pH and an ensuing conformational change in HA that leads to the hemifusion of the outer membrane leaflets of the virus and endosome, the formation of a stalk between them, followed by pore formation. Thus, the fusion machinery is an excellent target for antiviral compounds, especially those that target the conserved stem region of the protein. However, traditional ensemble fusion assays provide a somewhat limited ability to directly quantify fusion partly due to the inherent averaging of individual fusion events resulting from experimental constraints. Inspired by the gains achieved by single molecule experiments and analysis of stochastic events, recently-developed individual virion imaging techniques and analysis of single fusion events has provided critical information about individual virion behavior, discriminated intermediate fusion steps within a single virion, and allowed the study of the overall population dynamics without the loss of discrete, individual information. In this article, we first start by reviewing the determinants of HA fusogenic activity and the viral entry process, highlight some open questions, and then describe the experimental approaches for assaying fusion that will be useful in developing the most effective therapies in the future.

## 1. Introduction

Influenza virus is a member of the *Orthomyxoviridae* family and is classified into three types; influenza A, influenza B, and influenza C [1,2]. Influenza A virus comprises 16 hemagglutinin (HA) and 9 neuraminidase (NA) subtypes that have been determined by the distinct antigenicity of each of these proteins [3]. All subtypes of influenza A have been found to commonly infect avian species, which is thought to be the natural reservoir [4]. In addition, particular subtypes have also been isolated from pigs, horses, and humans, with the H1N1 and H3N2 subtypes currently circulating in the human population [5]. Historically, H2N2 viruses have also circulated widely in humans. Seasonal outbreaks present both a significant economic burden as well as having a major impact on public health, with an estimated 250,000 fatal cases per year [6]. Influenza is also responsible for the most devastating pandemic known to humans, with an estimated 50–100 million deaths occurring in the 1918 outbreak [7]. Seasonal outbreaks, coupled with the potential for a new pandemic, have promoted increased study of the determinants of viral pathogenesis and transmission, and much insight has been gained in our understanding of influenza infection—yet there are still many unanswered questions. This review will focus on determinants of HA-driven viral entry and membrane fusion, highlight open questions, and describe recently developed experimental approaches to study influenza virus entry and fusion.

**Figure 1:**
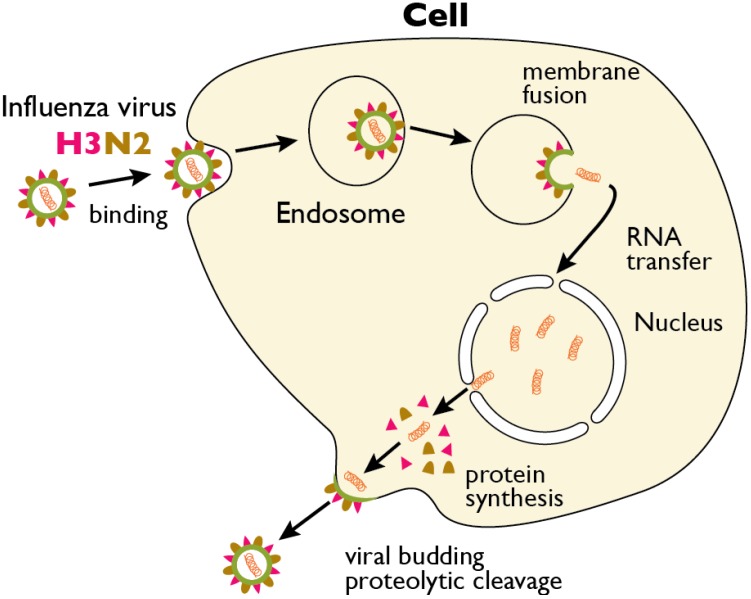
The replication cycle of influenza virus. Influenza virus binds to sialic acid groups present in the glycocalyx of the plasma membrane (glycocalyx omitted for clarity). The bound virus is subsequently endocytosed. Hemagglutinin (HA) is color-coded in red; Neuraminidase is color-coded brown. Ion channels and lipids are color-coded green. During maturation of the endosome, the pH drops, initiating the fusion of the viral envelope (green) with the endosomal membrane (gray) and the release of the viral RNA (orange) and viral proteins into the cytosol. The viral RNA traffics to the nucleus, where replication takes place. Newly formed viral RNAs are exported to the cytosol where they assemble with new virus structural proteins, which are packaged together at the plasma membrane, and bud off to form new virions.

An overview of the influenza replication process is shown in Figure 1. Influenza infection is initiated by the viral hemagglutinin (HA) binding to sialic acid receptors on the surface of the host cell. While the exact nature of HA-sialic acid interactions is complex, it is widely appreciated that the human-adapted HA subtypes preferentially bind to the α(2,6)-sialic acid linkage, whereas the avian-adapted HA subtypes preferentially bind to the α(2,3)-sialic acid linkage. This difference is thought to be a key determinant for host tropism [8]. A mutation in as little as one amino acid in the receptor binding domain can control the receptor specificity, increasing the likelihood of transmission to a new host [9,10]. 

Once bound, influenza enters the host cell by endocytosis. The internalization of influenza virus is not a simple process and can be highly cell-type dependent. Viruses have been shown to enter cells by both clathrin-dependent and clathrin-independent endocytosis [11,12,13,14], as well as by macropinocytosis [15,16]. Virus internalization is linked to the activity of receptor tyrosine kinases and other signaling molecules [17,18,19], and may require co-receptors that remain to be identified [20,21]. In polarized cells in particular, a dynamic actin cytoskeleton is critical for internalization [22,23]. Following internalization, the virus is trafficked through the endosomal network [12,24,25], with recent data suggesting that significant vesicle-vesicle fusion at the microtubule-organizing center occurs prior to virus-endosome fusion [26]. The acidic environment of the endosome triggers conformational changes in HA that expose the fusion peptide, allowing for viral-endosomal fusion [27]. Exposure to low endosomal pH is also necessary for release of the individual viral ribonucleoproteins (vRNPs) from the matrix (M1) protein, via the activity of the M2 ion channel found in the viral envelope [28,29]. Once the viral and endosomal membranes have fused, the viral genome and associated proteins are released into the cytosol, the vRNPs travel to the nucleus, and viral replication ensues [30,31]. 

The viral HA is intimately involved in two key steps in the influenza replication cycle, one being host cell binding and the other being viral-endosomal fusion. The focus of this review is HA-mediated membrane fusion, as it applies to influenza in humans. However for the HA protein to carry out this essential fusion step during viral entry, it must first undergo an important priming step, which occurs by proteolytic cleavage. Thus, we begin this review by considering the proteases that are needed for priming HA.

## 2. Proteolytic Cleavage of HA

### 2.1. Overview of HA Cleavage by Host Proteases

**Figure 2:**
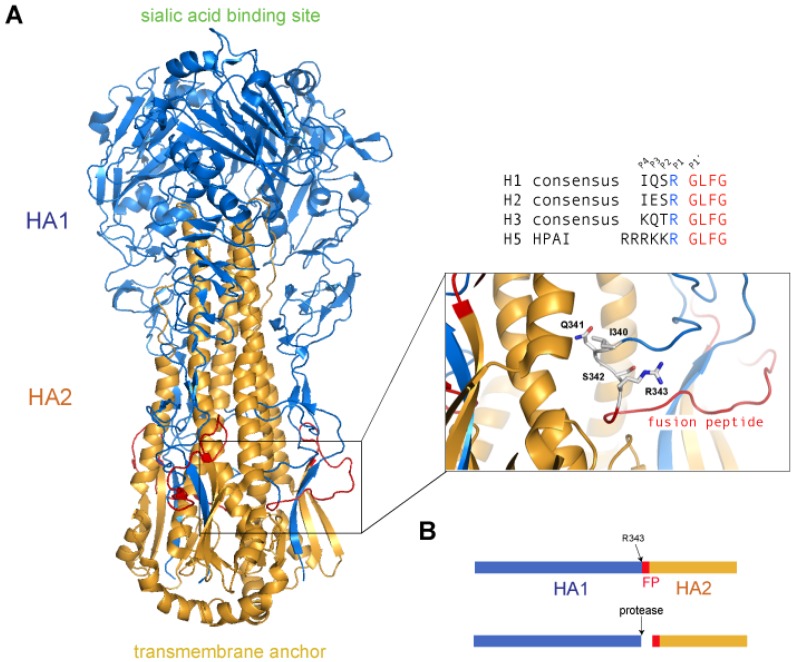
Cleavage of the HA precursor (HA_0_) primes influenza HA for fusion activation. (**A**) Structure of the non-cleaved, trimeric A/South Carolina/1/18 HA (H1N1) (PDB code 1RD8) [35]. The cleavage site region is enlarged (inset) with the side chains of the cleavage site residues (IQSR) shown. Equivalent cleavage sites are shown to the right for human H1-H3 and H5 highly pathogenic avian influenza (HPAI), along with the P4-P1’ positions recognized by the protease (**B**) Schematic diagram of HA. Upon cleavage by a host cell protease, the HA precursor is divided into two functionalsubunits, an HA_1_(receptor-binding) domain and an HA_2_ (fusion) domain. The newly created N-terminus of the HA_2_ subunit is the first residue of the fusion peptide (FP), which is exposed upon subsequent conformational changes and activation of HA triggered by the low pH of the endosome.

The viral hemagglutinin is synthesized as a fusion-inactive precursor in order to prevent premature fusion and/or HA activation throughout the secretory pathway [32,33]. HA must therefore be cleaved by host proteases in order to gain its fusogenic properties. Figure 2 provides an example of a complete HA protein and a corresponding residue map highlighting the cleavage sites. Cleavage occurs at the C-terminal end of a single basic residue for all HA subtypes. However, the cleavage site region differs amongst the HA subtypes, which in part, determines the degree of virulence. Highly virulent strains, such as highly pathogenic avian influenza (HPAI), contain a polybasic sequence at the cleavage site that allows for intracellular cleavage by ubiquitous, subtilisin-like proteases, such as furin [34]. In this case, since the host protease responsible for cleavage activation is ubiquitous, the HPAI strains are not restricted to a particular tissue, which is thought to be a primary determinant for the increased virulence. Low virulence strains (low pathogenicity avian influenza, LPAI) contain a monobasic cleavage site that is dependent on cleavage by trypsin-like, serine proteases that are either secreted into the extracellular space, or reside at the plasma membrane. The tissue tropism of LPAI is, in part, restricted by the localization of these proteases. Although not well characterized in avian species, the localization of equivalent proteins in humans is considered to be mainly in the respiratory tract. There remains, however, uncertainty regarding which proteases are key determinants for activation of LPAI HA, but a better understanding has been gained through recent insights in the proteolytic activation of HA, and is discussed below. 

#### 2.1.1. HA Cleavage by Transmembrane Serine Proteases (TTSP’s)

The hallmark discovery of certain transmembrane serine proteases having the ability to cleave and activate HA from the human-adapted subtypes has drawn much interest in this field of study. The TTSP family members TMPRSS2 and human airway, trypsin-like protease (HAT) have both been shown to facilitate trypsin-independent spread of influenza virus *in vitro* [36,37,38]. With both proteases being expressed in the human lung, they show a high potential to be involved in influenza infection *in vivo* [39]. Since the discovery of these two proteases, other members of the TTSP family have been determined to cleave and activate the viral HA. Chaipan *et al.*, demonstrated that TMPRSS4 has the ability to cleave and activate the influenza HA from the 1918 pandemic strain [38,40]. Okumura *et al.*, demonstrated that MSPL and a splice variant, TMPRSS13 have the ability to cleave and activate the HA from HPAI strains, which offers an alternative to cleavage by furin [41]. Although no definitive evidence yet supports their involvement in influenza virus infection *in vivo*, it is clear that these proteases could play a role in infection.

#### 2.1.2. HA Cleavage by Secreted Serine Proteases (TTSP’s)

A number of secreted, trypsin-like proteases have been found to cleave and activate the influenza HA. Proteases such as cellular trypsins, porcine mast cell tryptase, tryptase clara, and tryptase TC30 have been shown to cleave and activate HA from the H3 subtype (H3N2) [42,43]. However, it was determined that human mast cell tryptase is incapable of activating the influenza HA [42]. The blood proteases plasmin, urokinase, plasma kallikrein, and thrombin have also been found to cleave and activate HA [44]. Investigation of HA cleavage of a number of subtypes and strains by these blood proteases revealed both variability of cleavage within a given subtype and variability in the subtypes that each protease cleaved. This is intriguing, since each of the subtypes and strains tested contain a monobasic cleavage site with little variability in the cleavage site region for a given strain. Co-infecting bacteria have also been investigated for their role in HA activation, since they may provide an additional source of HA-cleaving proteases. Rott *et al.*, demonstrated that certain strains of *S. aureus* secrete a protease capable of activating HA and that inoculation of this protease along with influenza resulted in an increased virulence *in vivo* [45,46]. An important prospect for future work would be to extend this study to determine if any other commonly found co-infecting bacteria provide an additional source of HA-cleaving proteases. Overall, it is still unclear whether these proteases are present in the respiratory tract in their active form during the time of infection, but is evident that they have the ability to activate HA from the human-adapted subtypes, and therefore may play a role in influenza infection *in vivo*.

### 2.2. Other Factors Contributing to HA Activation and Function

Along with HA, the viral neuraminidase (NA) resides on the viral membrane and plays a pivotal role in infection. The viral NA is a glycosidase that functions to cleave sialic acid from both the glycans attached to HA and the glycans on the host cell surface, promoting release of the virion. In addition to release of the virion from the host cell, the enzymatic activity of NA leads to an enhanced infectivity. Su *et al.*, demonstrated an NA-dependent enhancement of fusion that is most likely due to desialylation of either the producer cell or the virion itself [47]. In addition, Reed *et al.*, demonstrated that the enzymatic activity of the H5N1 NA increased the pH range of HA fusion, where HA fusion was observed at a pH of 0.4 units above the pH of fusion in the absence of NA [48]. The viral NA has also been found to be involved in HA cleavage activation of particular influenza strains. Goto *et al*., demonstrated that the A/WSN/33 (H1N1) NA has the ability to sequester plasminogen, and so activate HA [49]. Further investigation determined that a C-terminal lysine and the lack of an N-linked oligosaccharide at position 130 of NA permitted sequestering of plasminogen [50,51]. The sequestered plasminogen is then activated by unknown means. The NA of the 1918 pandemic strain was also found to facilitate trypsin-independent activation of HA [52], but was later found to be cell specific and plasminogen-independent [40]. However, the mechanism by which the NA from the 1918 pandemic strain facilitates trypsin-independent HA cleavage remains undetermined. 

Another interesting aspect to HA cleavage activation is the observation that C-terminal residues of the HA_1_ subunit are cleaved upon activation of HA. Garten *et al.*, determined that a virion-associated carboxypeptidase was responsible for cleavage of the HA_1_ C-terminal residues [53]. However, cleavage of the HA_1_ C-terminal residues was found not to be critical for HA fusion, where HA with the full-length HA_1_ retained its haemolytic activity [54]. 

### 2.3. Molecular Determinants of HA Cleavage

The molecular determinants of HA cleavage are still largely unclear beyond the understanding that the proteases involved are most likely trypsin-like, serine proteases. However, through analysis of the studies conducted on HA cleavage, it has become apparent that there may be other factors besides the cleavage site residue itself that play a role in HA activation. Along with the variability observed with both the blood and bacterial proteases, Sun *et al.*, demonstrated that a natural mutation in the P2 position of the A/WSN/33 HA is a major determinant for cleavage by plasmin [55]. Optimal cleavage of the A/WSN/33 HA by plasmin was also demonstrated by Kido *et al*., where large differences were observed when comparing the plasmin cleavage efficiency of A/WSN/33 HA to other HA subtypes [42]. These studies revealed that the cleavage site flanking regions, to some degree, play a role in HA cleavage by host proteases. Analysis of HA cleavage by the blood proteases reveals that there may be a cleavage variability within a given strain that goes beyond the cleavage site region, since for the most part, the cleavage site amino acid sequence is identical for strains within a given subtype. This poses a challenging question to understand the molecular determinants of HA cleavage, but may be necessary to understand in order to determine the key host-proteases that are involved in HA activation. 

## 3. Influenza Entry Processes

### 3.1. Overview of the Roles of HA in Viral Entry

Proteolytic cleavage of HA is an important precursory step in the infection cycle that imparts new influenza viruses with the capacity to infect other cells. Within the HA protein, the cleavage site separates the two distinct subunits (HA_1_ and HA_2_) that control the essential operations of the viral entry process. HA_1_ contains the binding domain that permits the virus to attach to sialic acid receptors present on the host plasma membrane and initiates endocytosis. Once inside the endosome, HA_2_ then controls fusion between the viral membrane and the endosomal membrane, ushering the delivery of viral RNA to the host cell’s cytosol where it is trafficked to the nucleus. 

Influenza binding and fusion have been extensively studied, yet a number of questions remain from both a fundamental and practical point of view that are difficult to answer using standard experimental approaches. The most prevalent experimental techniques examine a collective population of virus, so-called ensemble-based approaches, which limit the information one can retrieve from the data. These limitations become especially pronounced when multiple processes occur within particular protein machinery, such as HA. HA controls both the binding and fusion of the virus with the host cell and so these processes must be decoupled from each other experimentally to assess changes due to strain variation or other factors, and to determine any synergy between these processes during infection. By examining binding and fusion in individual viruses (the single particle approach), unique information about each separate process can be retrieved from both the stochastic behavior of individuals and through examination of the aggregated population dynamics. These advantages create an impetus for the development of new single particle experimental assays for fundamental virology studies. 

There are practical reasons to develop assays that decouple these processes as well. As the HA protein is presented on the surface of the virus, it is an important target for anti-viral mediations. Many influenza antibodies and compounds developed in the past targeted the most exposed regions of the protein. Antibodies against the HA_1_ portion of the virus [56,57] can disrupt the binding process. Other antibodies are thought to interact with other exposed areas of HA, supported by crystallographic evidence and measured shifts in pH of fusion relative to wild type HA [58]. While the rapid mutation rate of HA_1_ makes protection by these antibodies short-lived, recently several studies have shown that antibodies can be made against the conserved region of the HA stem that presumably disrupts the conformational change that must occur for fusion [59,60,61,62,63]. The expectation is that this type of antibody would not only confer long-lasting protection, but also protect against a number of different strains. 

In the case of a well-known fusion inhibitor, *tert*-butylhydroquinone (TBHQ) [64], single particle assays could have assisted in defining where it interrupts the entry process. An excellent review of this fusion inhibitor is given by Modis [64]. TBHQ compound was originally predicted to bind near the fusion peptide by *in silico* analysis [65], but fusion studies with H1 and H3 subtypes by ensemble lipid mixing and leakage assays showed that it had limited anti-fusogenic activity for H1 [66], while it afforded some protection from infection by H3 subtypes [65]. Later crystallographic studies showed that TBHQ does not bind near the fusion peptide, but in a hydrophobic pocket that can impede the conformational change required for fusion [67,68]. While crystallography will still be required for future compounds to determine the exact binding interaction with the HA, direct methods of assaying fusion and its intermediate steps can assist in defining how the interaction disrupts the fusion event itself. 

A few novel fusion-inhibiting compounds have become available recently. Several compounds appear to impede an early step in the influenza infection process [69,70], while a novel cholesterol conjugated, anti-fusogenic peptide, localized to endosomal membrane, is believed to prevent fusion by blocking the second critical conformational change that drives the membranes together [71]. New experimental techniques are key to directly characterizing the steps where compounds impede infection and for quickly screening new potential compounds. Such assays will speed up the development of new anti-fusogenic compounds and increase our understanding of the molecular level mutations of the HA_2_ region that impact the fusion process and drug interactions [72]. 

A shift in the paradigm of studying virus fusion from an ensemble perspective to studying single virion fusion events offers exciting possibilities to answer many subtle questions about fusion, develop new characterization and screening tools, and determine efficacy of new classes of anti-viral compounds. This kind of approach has only become possible in recent years with the advent of highly sensitive imaging techniques, microfluidic fluid handling, and robust membrane materials. In the following sections, we describe the features of the individual virion imaging (IVI) approach, contrast them with traditional approaches for studying viral entry processes, and summarize the recent advances made using IVI.

### 3.2. HA Mediated Fusion Mechanism

**Figure 3:**
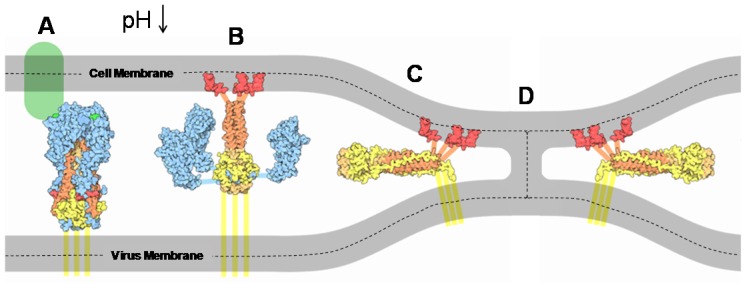
HA-mediated membrane binding and fusion between the viral and endosomal membranes. (**A**) HA_1_ (blue) binding to a moiety containing sialic acid group on the plasma membrane (light green); (**B**) After a reduction in pH in the endosome, HA_2_ undergoes a conformational change that drives the fusion peptides (red) into the host cell membrane; (**C**) A further conformational change brings the outermost leaflets of the opposing membranes together to form a stalk (**D**), where it is thought to be the action of several fusogenic HA_2_ working in concert. The dashed lines divide the upper and lower leaflets of the membranes for clarity. (Figure adapted from the Protein Data Bank [89]). Eventually the stalk collapses to form a pore (not shown).

The actual transfer of viral genetic material to the cytosol requires the fusion of the viral membrane with the endosomal membrane and the formation of a fusion pore, through which the genetic material exits the viral capsid. Membrane fusion proceeds through a series of distinct steps following endocytosis and that are controlled by the HA_2_ portion of the hemagglutinin protein [73,74,75,76,77]. These steps are outlined in Figure 3, starting with the binding step of HA_1_ to the plasma membrane of the host cell. Activation of the fusion machinery within the HA_2_ occurs during the drop in endosomal pH during maturation. Acidification initiates a first conformational change of the HA_2_ leading to the exposure of the fusion peptide [65,77,78,79]. When the endosomal membrane is in the vicinity, this hydrophobic peptide inserts into the interior of this opposing membrane, becoming anchored there due to strong hydrophobic interactions with the lipid acyl chains [80]. Following this insertion step, it is generally believed that several HAs form a cluster, a so-called fusogenic unit [81,82]. The optimum number of HA units in this cluster is generally believed to be greater than one, but reports vary widely in the literature [72,83,84,85,86,87]. In the next step, the proteins within the fusogenic unit undergo a further conformational change, bending back at a hinge point to drive the two opposing membranes together and dehydrate the space between them. The merging of the two outermost leaflets of the opposing membranes then forms a “stalk” where mixing of the lipids from the outer leaflets is commonly referred to as hemifusion [88]. At some later time point the stalk, or a section of the membrane near it, ruptures to form a fusion pore through which the viral RNA can escape into the host cell cytosol. 

### 3.3. Open Questions in Fusion

While membrane fusion mediated by HA has been a well-studied subject, a number of key questions still remain unanswered. It is still not clear how many HA trimers are required to mediate fusion, or if the number varies depending on the viral subtype and strain. Part of the difficulty in assigning this value stems from the manner, conditions, and analysis by which it is determined [87]. Improvements in imaging techniques and single particle approaches will shed light on this area. 

A second open area for investigation is discovering the rationale for HA carrying out both binding and fusion processes; *i.e.*, if a single HA carries out both processes, and if this is a necessary condition of viral entry? One hypothesis that has been put forth to rationalize binding/fusion function in one protein is that upon binding, the energy landscape may change such that the fusion conformational states are more energetically favored [90,91]. Furthermore, this favorable energy state may only be achieved when the protein binds the right target and in this way ensures that fusion is triggered only when the proper target is in place [92]. This hypothesis is supported by studies where impeding binding with sialyllactose abolished fusion at all pH values. One explanation consistent with this result is that binding is a necessary precursor step that allows HA to enter a fusogenic conformation [93]. It has also been shown that the HA_1_ subunit, and the transmembrane and cytosolic domains of HA_2_ are not required for fusion pore opening [94]. Clarification of the role of HA in both processes may be critical for determining if a single anti-fusogenic drug can prevent infection or if prescribing a cocktail or multi-purpose drug is required to cover both processes. 

A third area of importance is assessing the impact of membrane physico-chemical properties and the role of lipids and membrane constituents on fusion kinetics. Nussbaum *et al*. [95] report that both cholesterol and receptor in the host membrane is required for fusion with zwitterionic membranes but not for negatively charged (phosphoserine-containing) or phosphoethanolamine-containing membranes. Chernomordik *et al*. [96] studied fusion as a function of triggering pH, comparing liposomes with curvature altering liposome constituents, lysophosphoatidylcholine, oleic acid, and found that as the triggering pH was lowered, the composition of the liposomes was decreasingly important, *i.e.*, the increasing number of activated HA that can participate at lower pH’s eventually outweighed the impact of the particular lipid shape and its influence on membrane bending. Work by Bailey *et al.* [97] extended this work and examined the impact of cardiolipin as well, and concluded that these membrane dopants can alter pH-dependent fusion, especially in the moderately acidic range. In contrast, others report that material properties of host membranes may not be critical for fusion [66]. In this study, fusion was assessed using virus-liposome mixing assays covering a range of compositions where membrane curvature and void stabilization was examined. No correlation was found between hemifusion rates and membrane rupture tension. 

Razinkov and coworkers [98] examined the impact of membrane constituents on the formation and growth of fusion pores during fusion of influenza with planar black lipid membranes (BLMs) using an electrochemical approach to monitor the stochastic flickering of the fusion pore. In this work the impact of cholesterol and its analogs and various sphingolipids were examined and found to strongly influence the growth of pores. Open questions also exist about the composition of the fusion pore. Chernomordik *et al.*, propose a lipid-lined hemifusion pore with restricted lipid diffusion just prior to pore formation [99]. Bonnafous *et al.*, propose the formation of a small protein lined pore for host cell and lipid lined pore for viral membrane just before hemifusion commences [100].

The role of lipid rafts has been suggested to be important for organizing viral proteins prior to budding, but less is known about the role of rafts in viral entry. HA inherently associates with common lipid raft constituents like cholesterol and sphingomyelin and it has been shown that deleting the raft-associating part of HA reduces fusion over 50% [101]. 

## 4. Studying HA Fusion Kinetics

### 4.1. Overview

Among the 16 different HA proteins known, there are marked variations in binding and fusion properties. New experimental approaches may enable characterization of entry processes in ways that were not possible previously because binding and fusion could not be completely decoupled. Characterization of the fusion kinetics of different strains of influenza is beneficial for understanding the fusion process and assessing strain to strain variations [102]. These include determining if strain variation is manifested as changes in fusion and if these changes correlate to increased infectivity, assessing the importance of the fusion step in pandemic strain emergence, determining the influence of membrane chemistry on fusion and evaluating its connection to systemic infection, and establishing the efficacy of newly developed anti-fusogenic antibodies and anti-viral drugs. 

*In vitro* fusion assays employed to characterize virus fusion should mimic the *in vivo* endosomal environment as closely as possible. Ideally intact virions should be used to preserve the natural features of the virus, which may play as yet unknown supporting roles in fusion. In addition, assays with fast triggering and data acquisition capabilities with suitable time resolution that does not obscure or influence the processes under study is paramount to acquiring the most quantitative data [103]. To begin this section, we first review traditional fusion assays and their limitations, and then describe the state-of-the-art fusion assays enabled by modern technologies. 

### 4.2. *In Vitro* Ensemble Fusion Assays

The first *in vitro* strategies for studying virus fusion to membranes were ensemble or bulk assays [104]. These initial assays reported either membrane mixing between the virus and the cell membrane or content release of the virus; but not both at the same time [105]. Content mixing assays typically employed vesicles with encapsulated dye that upon fusion with either intact virions or reconstituted viral envelopes release their contents and give rise to a change in fluorescent signal. Two common approaches are the release of a calcium indicator dye that fluoresces when exposed to the surrounding buffer or changes in fluorescence due to dequenching of internal dye or fluorescence energy transfer (FRET). In membrane mixing assays, virus fusion is typically reported by changes in fluorescence resulting from fluorescence dequenching within the membrane upon fusion [106], or FRET between fluorophore pairs residing in the membrane [105]. 

In the most direct measurement of membrane mixing and associated kinetics, intact viral membranes are first labeled with fluorophores until the fluorescence signal is quenched. Then, the labeled virus is mixed in a cuvette with unlabeled host cell mimics containing the viral receptor, such as liposomes or ghost cells, and a baseline florescence signal is obtained in a fluorimeter. To initiate fusion, a small amount of acid is added to the cuvette while the sample is rapidly mixed. The temporal change in the fluorescence signal is collected as the viruses fuse with the host membranes and the fluorophores originally in the viral membranes become diluted and dequench. Alternatively, a FRET method can be used to avoid labeling the virus itself by creating liposomes containing both fluorophores of a FRET pair that separate when virus fuses to vesicle.

From the change in the fluorescence signal in either approach, some information about the kinetics of virus fusion can be obtained. These approaches characterize the overall rate from the binding to the hemifusion step, determined over an ensemble population of virions within the cuvette. Many studies of virus fusion to date have been conducted using this type of assay [96,104,106,107,108,109,110] and a great deal of what is known about virus fusion has been learned using this ensemble approach. However, there are several limitations that have restricted the amount of information that can be collected from these assays. First, virus fusion is stochastic and thus only averaged kinetic information is obtained from these assays, which can obscure intermediate steps [103]. Second, since individual events cannot be observed in this assay, viral binding and fusion events cannot be distinguished visually making it difficult to study either processes individually. To circumvent this limitation, these assays can be conducted at cold temperatures to bind viruses first and then trigger with acidic buffer to decouple binding and fusion processes kinetically from each other [81]. An instantaneous pH change from neutral to acidic is ideal to trigger virus fusion at the same time point at a uniform pH value. Asynchronous triggering of events masks the magnitude of the pH dependence of fusion [92], which may be an important criterion when assessing infectivity. Therefore, due to the finite volume of the cuvette, rapid mixing of contents is required to quickly distribute the acid throughout the cuvette, but this rapid mixing leads to shearing, which can interrupt virus binding and does not mimic the quiescent environment inside an endosome. A third important consideration is that the curvature of the two opposing membranes is opposite of that inside the endosome. It is unclear if this non-native geometry could result in membrane bending energies that alter the kinetics or pathway of fusion of the membranes. Finally, monitoring pore-opening kinetics is difficult to conduct simultaneously with membrane hemifusion in this ensemble approach. Many of these drawbacks can be overcome using IVI approaches and alternative cell membrane mimics with planar geometry.

### 4.3. Early Individual Virion Imaging of Virus Fusion to Cell Membrane Mimics

Recognizing ensemble assay limitations, investigators moved to studying single event virus fusion using direct imaging of virions, reconstituted viral envelopes (virosomes), or HA-expressing cells interacting with other cells or cell membrane mimics. Several fusion studies of intact virus to erythrocytes [111] or individual human erythrocytes to fibroblasts expressing the influenza virus hemagglutinin were reported [112,113]. These assays employed a flow chamber mounted to a microscope stage. Fusion was triggered by rapid acidification of the flow chamber and fusion monitored by a fluorescence increase due to redistribution of fluorescent dyes between either membrane or cytoplasmic compartments of fusing cells. Significant heterogeneity in lag times for events was reported, which could be in part due to asynchronous initiation of fusion, a point we will return to later. 

Niles and Cohen [114], used a video-epifluorescence microscope setup to study individual virions fusing to a planar BLM formed across the orifice of a Teflon support [115] positioned within the field of view of a microscope. In the execution of this assay, fluorescently-labeled, quenched viruses were loaded into a micropipette tip which was positioned in one side of the Teflon chamber already at the desired fusion pH. To coordinate the triggering of fusion, virus solution (at neutral pH inside the micropipette) was gently expelling near the acidified BLM surface. Virions contacting the BLM, either immediately bound to it or fell out of the field of view quickly. Bound viruses could then undergo fusion with the BLM. A video camera recorded the fluorescent images of individual fusion events, detected as single dequenching events. These images were later processed to obtain hemifusion kinetic parameters. 

The Niles and Cohen [92] individual virion fusion technique showed that receptor binding alters fusion kinetics. In the presence of receptor, the kinetics followed Markovian behavior characteristic of Poisson process described by a rate constant defining the jump between distinct states. Fusion triggered in the absence of receptor followed non-Markovian behavior with no characteristic rate parameter. This clear distinction in fusion kinetics was made possible by (1) decoupling of binding and fusion processes; (2) temporal synchronization of fusion initiation; and (3) statistical analysis of the individual fusion events. While it was known from previous ensemble studies that gangliosides increase fusion rate [116,117,118], the single virion approach of Niles and Cohen could quantitatively assess changes in rate and provide additional information about the kinetic pathway [92,93]. These measurements were corroborated by electrical conductance measurements [119]. Later imaging assays that combined lipid mixing, contents mixing, and electrical conductance measurements in one assay provided important experimental details on the intermediate steps leading to pore formation and that it might proceed by a series of small pores forming initially [120]. 

Importantly, these first experiments pioneered the approach of direct visualization of individual influenza fusion events and using planar bilayers (in the form of BLMs) as the cell membrane mimic. However, the limited sensitivity of the equipment and the fragility of BLMs restricted the broader applicability of this method at the time. Nonetheless, these studies ushered in a new stochastic approach for studying virus fusion, which laid the critical groundwork for IVI assays of today.

### 4.4. Stochastic Fusion Assays

Building upon the previous assays and with the advent of increasingly better technology for implementing single particle studies, fusion studies involving cell-cell fusion, virosome-cell fusion, and virus-cell fusion at the individual event level continued, targeting a variety of viruses [86,121,122]. Studies using reconstituted vesicles of HA allowed researchers to assess the impact of HA density on fusion. One study examined the impact of various forms of HA on fusion, reconstituting both HA_0_(nonfusogenic form) and HA_1,2_ (fusogenic form) into vesicles [86]. This study suggested that coordination of multiple HA trimers may not be necessary for fusion and that HA binding to its receptor might actually interfere with fusion, supporting that individual HA’s may not carry out both binding and fusion processes even though they are able to do so. A recent systems biology approach of analyzing fusion data supports that HA’s carry out separate functions during viral entry [87].

### 4.5. Single Virion Fusion Using Total Internal Reflection Fluorescence Microscopy

**Figure 4:**
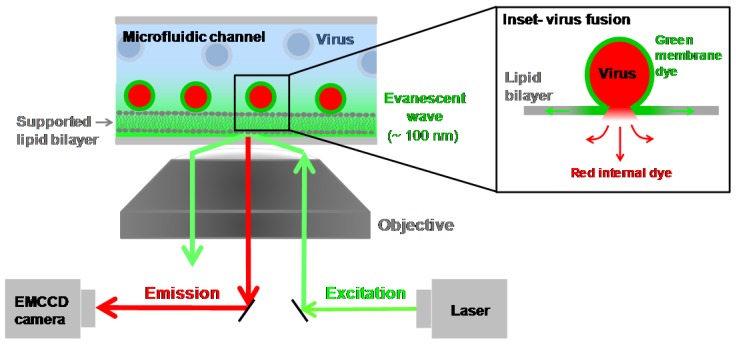
Total internal reflection fluorescence microscopy integrated with a microfluidic device. A coherent laser source can be steered through an inverted microscope objective at or above the critical angle for total internal reflection as dictated by Snell’s law, generating an evanescent wave at the glass-buffer interface. A supported lipid bilayer adsorbed to the walls of the microfluidic device will reside within this evanescent wave. Fluorescently-labeled viruses bound to the supported bilayer will be excited and emit a red signal. The emitted light is sent back to a sensitive camera for imaging. The addition of a second laser line or more allows multiple fluorophores to be monitored simultaneously, for example, one to mark the viral membrane and another to mark the internal contents. Note that the size of the virus with respect to the bilayer is not drawn to scale. The bilayer is ~4 nm thick, while the virus is typically ~100 nm in diameter. (Inset) Upon acidification of the microfluidic channel, virus fusion commences. A two color virus labeling scheme distinguished the hemifusion step (green) from pore formation (red).

Today’s sophisticated electronics capable of single molecule fluorescence detection, microfluidic approaches for fluid handling, and new strategies for creating robust membranes have made single virion fusion studies even easier to implement with intact virions. This is an important feature of these assays, as it is not entirely clear that HA-expressing cells of reconstituted virosomes would behave the same way that a whole virus with its intact viral membrane and secondary proteins does. Recent work using intact virions in stochastic assays has created a wealth of new knowledge about fusion kinetics by providing information on the kinetics of intermediate steps of the fusion mechanism [123] and information about the rate of acidification of the internal contents of the virus during fusion [124]. These new assays employ total internal reflection fluorescence microscopy (TIRF) [125], a surface-specific technique that illuminates about a 100 nm thick layer from the interface of a change in refractive index, such as between glass and aqueous solution, and greatly facilitates distinguishing bound viruses from those in the bulk solution. Because TIRF is a surface-specific technique, it is compatible with planar platforms like microfluidic devices. Microfluidic devices that have their walls coated with solid-supported lipid bilayers have been employed for numerous bio-analytical applications aimed at mimicking the cell surface. Supported lipid bilayers are robust materials that are easy to fabricate, their physico-chemical properties can be tailored, and the incorporation of membrane receptor molecules is straightforward [126,127,128]. The spacing between the bilayer and the solid support can be tuned using cushions as well [129,130,131]. These biomimetic TIRF platforms lend themselves well to single particle studies of vesicle rupture at surfaces [132]; two-dimensional diffusion of phospholipids [133,134], proteins [135], and virus-like particles [136] in planar bilayers; vesicle fusion mediated by SNARE proteins [137,138,139] and DNA hybridization [140]; and virus fusion [123,141], as illustrated in Figure 4. 

Acidification to initiate virus fusion in this platform is achieved by flowing an acidic solution through the microfluidic channel to exchange with the neutral buffer initially in the device. 

Intermediate fusion steps can be distinguished using a multi-color labeling procedure where the viral membrane is labeled with one color and the internal viral contents are labeled with another color [123]. This strategy enables one to monitor the hemifusion process separately from the pore formation process within a single virion and to examine their relative timescales. Hemifusion detection proceeds through a dequenching strategy, as was employed in previous ensemble assays, but here clearly marks the onset of the hemifusion event for an individual virion. Pore formation within the same virion is visibly detectable in this assay, through the release and subsequent diffusion of the internal fluorescent dye away from the fusion site. 

### 4.6. Data Treatment and Stochastic Analysis

Each individual fusion event occurs independently. From each fusion event, a number of critical pieces of information can be obtained. First, the lag time between acidification and hemifusion for that individual virion is marked by the time point at which the dequenching spike occurs. Second, information about the diffusion of the lipids at the fusion site can be obtained by monitoring the radial spread of the fluorescence following the dequenching spike. Diffusion coefficients can be determined from the data by fitting it to the solution of the two-dimensional Fick’s diffusion equation. Third, the lag time between hemifusion and pore formation for the same virion can be easily determined when using a two-color labeling strategy. Cataloging the lag times for each individual fusion event yields statistical data that can then be analyzed to determine the kinetics of fusion for a population. 

Within each fusion event, a number of intermediate steps occur between the initial conformational change of HA triggered by acidification and the creation of the fusion pore. The overall fusion process is a convolution of the various intermediate steps, each of which can be described as a Poisson process. The distribution of the lag times of a population can be modeled by a gamma distribution to obtain a shape, *N*, and rate parameter, *k*. When examining the hemifusion lag time distribution, *k* defines the hemifusion rate constant and *N* is interpreted to be the number of HA trimers working concertedly to bend and merge the membranes, as described in detail by Floyd *et al.* [123]. In this work, analysis of pore formation lag times for X:31 yielded a single exponential decay, indicating a one step process from the hemifusion state to the formation of the pore. 

### 4.7. Limitations of Current IVI Assays and Considerations for Future Improvements

While acidification by buffer flow exchange in microfluidic devices works reasonably well to trigger the conformational change of HA, a no-slip boundary condition at the membrane surface creates shear on viruses bound to the membrane. Therefore, the rate of buffer exchange must be low enough to minimize shearing viruses off the receptors and/or stretching of the protein conformations that could cause non-native fusion protein-proton interactions. This requirement creates an upper bound to how fast exchange can occur.

The dynamics of the protein conformational changes for some HA proteins are known to be on the millisecond timescale [142], so slow acidification by buffer exchange may present limitations in resolution of the technique. For the laboratory adapted strain, X:31, at the “optimal” fusion pH (~4.9) the protein conformational change is not the rate-limiting step in the fusion process [142]; however at suboptimal pH, the slow transition to the fusogenic conformational form of HA leads to slower kinetics and a decreased extent of fusion [142]. Slow acidification can also temporally spread initiation of fusion events across the field of view and possibly impart local pH gradients. These effects can limit the resolution of the data that can be obtained. 

In the future, more information could be garnered by labeling the viral contents, for example the viral RNA, so that unpacking of the viral caspid and release of the viral genome is confirmed. DNA/RNA labeling of non-enveloped viruses [143] and phages [144] has been demonstrated, but at present labeling the viral RNA inside a membrane-enveloped virion is still a challenge. Such an advance would not only aid in learning more about the fusion process, but allow for studies of RNA unpacking upon release and assist in identifying other potential targets for future anti-viral compounds.

## 5. Looking Ahead

With the establishment of these new approaches to study fusion, we anticipate that future studies will focus on a range of factors in virus fusion that have been difficult to examine in the past, continuing to fill in fundamental information that will inform the rational design of antiviral therapeutics. In additional to fundamental fusion studies, these new tools offer better ways to characterize variations in fusion behavior among various virus strains, lab-adapted varieties, and clinical isolates, and the action of anti-fusogenic compounds acting on them. Novel therapeutics such as protease and fusion inhibitors and cross-neutralizing antibodies that impede the fusion process may be directly studied in these platforms as well. Furthermore, it may be possible to also study endosome-specific factors in fusion, such as the impact of various lipids and enzymatic reactions that modify lipid species as the endosome matures, by replicating these reactions within the *in vitro* platform. 
